# Finite element analysis of adhesive endo-crowns of molars at different height levels of buccally applied load

**DOI:** 10.1177/1758736012455421

**Published:** 2012-08-03

**Authors:** Istabrak Hasan, Matthias Frentzen, Karl-Heinz Utz, Daniel Hoyer, Alexander Langenbach, Christoph Bourauel

**Affiliations:** 1 Rheinische Friedrich-Wilhelms University of Bonn, Bonn, Germany; 2 Department of Periodontology, Operative and Preventive Dentistry, Rheinische Friedrich-Wilhelms University of Bonn, Bonn, Germany; 3 Department of Prosthodontic, Preclinical Education and Material Science, Rheinische Friedrich-Wilhelms University of Bonn, Bonn, Germany

**Keywords:** endo-crown, monobloc, primary abutment, loading position, equivalent von Mises stress, total equivalent strain

## Abstract

This study aimed to evaluate the biomechanical behaviour of adhesive endo-crowns and the influence of their design on the restoration prognosis when four loading positions are applied from the restoration–tooth junction. Two three-dimensional finite element models for the lower first molar were developed: endo-crown as a monobloc and endo-crown of a primary abutment and a full crown. Four crown loading positions were considered: 5, 6, 7 and 8 mm. A force of 1400 N was applied buccally on the middle of the mesiodistal width. No differences were observed for the two endo-crowns concerning restoration displacement and the distribution of equivalent von Mises stress and total equivalent strain. Shifting the position of the applied load to 8 mm resulted in an increase in the displacement from 25 to 42 µm and an increase of equivalent von Mises stress concentration at the tooth. The height of load application on the restoration has a significant role in the prognosis of endo-crowns.

## Introduction

The rehabilitation of severely damaged coronal hard tissue and endodontically treated teeth is always a challenge in reconstructive dentistry. Clinical concepts regarding the restoration of non-vital teeth are controversial and are often based on profuse and inconclusive empirical literature. The primary reason for reduction in stiffness and fracture resistance of endodontically treated teeth is the loss of structural integrity associated with caries, trauma and extensive cavity preparation rather than dehydration or physical changes in the dentine.^1–4^ Reduction of the tooth architecture results in increased cuspal deflection during loading, either continuous or cyclic, and delayed cuspal recovery following removal of the load.^5–7^ Therefore, the loss of structural integrity increases the occurrence of crown fractures and microleakage at the margins of restorations in endodontically treated teeth compared with ‘vital’ teeth.^4,8^ Additionally, it was argued that the lack of vitality greatly restrains the sensory feedback during peak loads and results in non-vital teeth being more prone to fracture.^9^

The classical approach for restoring endodontically treated teeth is to build up the tooth with a post and core, which have physical properties close to those of natural dentine, utilising adhesive procedures and placement of full-coverage crowns with a sufficient ferrule.^10–12^

The optimal modulus of elasticity for dental restorations has been discussed, the issue remains controversial.^12–15^ Rigid restorations that adhesively retained to the remaining tooth structure may offer good support and distribute the stress more uniformly; however, if the tooth is overloaded, a catastrophic failure may result, such as a vertical or deep root fracture.^16,17^ A more elastic restoration may bend under high loads, resulting in loss or failure of the restoration, but would leave the root intact for retreatment.^18,19^ The recent developments of adhesive techniques and ceramic materials facilitated the avoidance of possible operational errors during post space preparation, by introducing the so-called endo-crown.^20^

The advantage of adhesive restorations is that a macroretentive design is no longer a prerequisite if there are sufficient tooth surfaces for bonding. Minimally invasive preparations to preserve a maximum amount of tooth structure are considered to be the standard main goal for restoring teeth. Endo-crowns strictly follow this rationale owing to a decay-orientated design concept. This type of preparation consists of a circumferential 1.0- to 1.2-mm butt margin and a central retention cavity inside the pulp chamber and constructs both the crown and core as a single unit, that is, a ‘monobloc’.^20^ The monobloc foundation of this technique utilises the available surface in the pulp chamber to obtain stability and retention of the restoration through adhesive bonding. Moreover, dental computer-aided design/computer-aided manufacturing (CAD/CAM) systems realise the possibility of chair-side design and automatic production of these single-unit ceramic restorations. However, posterior all-ceramic CAD/CAM full crowns have been preferentially used as In-Ceram core crowns because of the high strength of the core ceramic.^21^ CAD/CAM fabrication of In-Ceram restorative parts is a multi-step process based on the prefabrication of blocks with an open-porous structure.^22^ CAD/CAM crowns out of MK II feldspathic bloc-ceramic gain sufficient strength only when bonded.^2,23^ Adhesive cementation increases the strength and the resistance of ceramics to fracture.^24–26^

Although endodontically treated molars restored with endo-crowns have been reported to be clinically successful,^9^ clinical and in vitro studies indicated more frequent problems with endodontically treated premolars restored with endo-crowns.^27,28^

In vitro studies^29,30^ were carried out in the Department of Conservative Dentistry of the Dental Clinic of the University of Bonn. In these studies, the strength and fracture resistance of two CEREC^®^ endo-crown systems were investigated in endodontically treated upper and lower molars. In total, 40 molars were treated with monoblocs and 47 were treated with endo-crowns as a combination of primary abutments adhesively cemented to a full crown. The restored teeth were subjected to a mechanical loading using a self-developed mini Zwick machine with cyclic loading of 800 cycles up to 1400 N. The cyclic loading of the molars resulted in seven failures of monoblocs and 31 for those with primary abutment.

Hence, the aim of this study was to investigate numerically the previously in vitro investigated two endo-crown restoration systems and to analyse their fracture resistance and fracture modes with the variation of the position of the applied load from 5 to 8 mm. The developed three-dimensional (3D) finite element (FE) models were based on the parameter setting of the above-mentioned previous studies.

## Materials and methods

**Figure 1.**
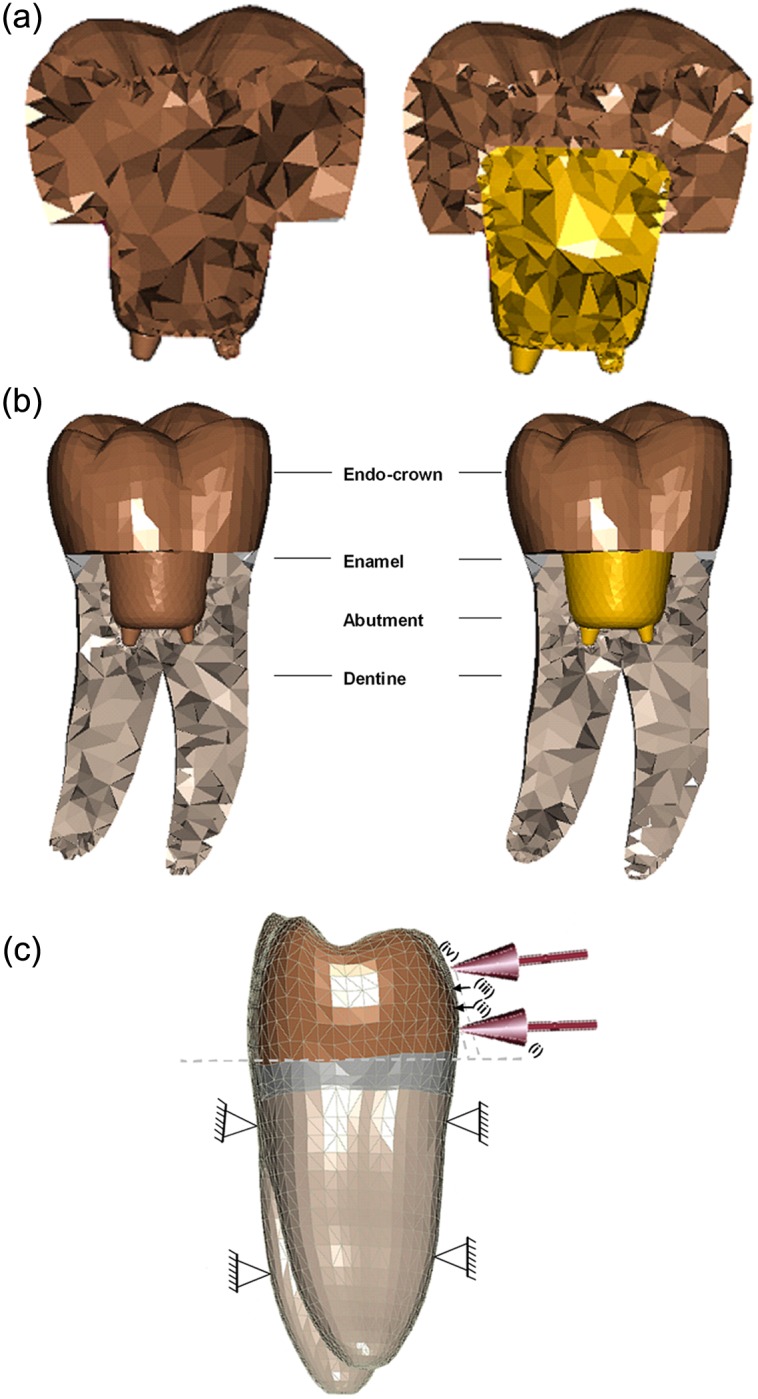
(a) Longitudinal cross section of the two endo-crown models. Monobloc (on the left) and endo-crown with primary abutment (on the right). (b) Longitudinal cross section of the numerical models describing their components. Monobloc (on the left) and endo-crown with primary abutment (on the right). (c) Configuration and boundary conditions of the numerical models with a force of 1400 N: (i) loading point is 5 mm from the restoration–tooth junction; (ii) loading point is 6 mm from the restoration–tooth junction; (iii) loading point is 7 mm from the restoration–tooth junction and (iv) loading point is 8 mm from the restoration–tooth junction.

Two 3D FE models for a lower first molar were developed: In the first model, the endo-crown was considered to be a monobloc that adhesively retained to the prepared tooth (Figure 1(a), left). In the second model, the endo-crown consisted of a primary abutment that adhesively retained to a final full ceramic crown; both adhesively retained to the prepared tooth as well (Figure 1(a), right). Since the adhesive film has an optimal contact with the prepared dentine on one side and the ceramic crown on the other side, an ideal continuity was considered for the numerical analysis. Furthermore, none of the investigated teeth in the previous experimental study showed a disengagement of the adhesively retained ceramic crowns from the dentine.

An idealised geometry of a lower first molar was selected for this study (Digimation Corp., St Rose, LA, USA). The models consisted of tooth roots, cervical remaining ring (about 1.5 mm) of the coronal portion and the endo-crown (Figure 1(b)). The models were generated using FE program MSC Marc/Mentat 2007 (MSC Software Corporation, Santa Ana, CA, USA) and tetrahedral element type (four nodes) that was selected for model generation. The total number of elements was 57,000 and 78,000 for monobloc and endo-crown with primary abutment, respectively. The preparation depth, position of crown projection in the root canals and the position of the finishing line were the average of the measured data from the radiographical documentation of the experimentally tested teeth. In so far, tooth and endo-crown geometries are idealised ones and differ by up to 20% in volume from real tooth or crown geometries. Consequently, the results are valuable for comparison between the groups tested here and should not be transferred to clinical practice without further discussion.

Material properties of the numerical models are illustrated in Table 1. Boundary conditions and loading nature of endo-crowns were based on the previous in vitro studies^29,30^ as follows:

1. In vitro, before the molar teeth were mounted in the permanent loading frame, the roots were embedded up to the cementoenamel junction in a resin. Accordingly, the embedded portion was fixed in three degrees of freedom in the numerical models.
2. After mounting on the loading frame, the teeth were loaded with a force of 1400 N. The range of vertical bite forces as described in the literature is between 320 and 847 N.^31^ During the in vitro measurements, a force magnitude was chosen to be much higher than the maximum force that was published in the literature. For this reason, 1400 N was selected to be the critical loading value for the experimental study. The force was applied perpendicular to the crown surface buccally at the middle of the mesiodistal width and about 5 mm above the restoration–tooth junction (for monobloc) and 8 mm (for the endo-crown with primary abutment, see Figure 1(c)). The same situation was considered for the numerical models.

**Table 1.** Material properties of the numerical models

Material	Young’s modulus (GPa)	Poisson’s ratio
Enamel^32^	41.0	0.30
Dentine^32^	18.6	0.30
Ceramic (VITABLOCS Mark II)^33^	62.0	0.23

The choice of this force magnitude was based on the cyclic loading of the experimentally tested teeth. Most of the endo-crowns were broken by applying this force magnitude.

In this numerical analysis, four loading heights were studied for both endo-crown designs, namely, 5, 6, 7 and 8 mm from the restoration–tooth junction. The reason for the investigation of the different heights of the point of force application was to determine the interrelationships between the position of the finishing line, the lever arm for force application and the design of the endo-crown.

## Results

### Tooth structure/root region

The obtained values for equivalent von Mises stress and total equivalent strain of model with monobloc were close to those obtained for the endo-crown with primary abutment. The distribution of the stress and strain was similar for both models. However, applying the load at different heights showed noticeable differences: Applying the force at 5, 6, 7 and 8 mm distance from the restoration–tooth junction resulted in equivalent von Mises stresses of 54–57, 60–63, 66–73 and 87–90 MPa and total equivalent strains of 2000–2300, 2500–3000, 3300–4000 and 3800–4200 µϵ, respectively. The ranges of the obtained stresses and strains stand for the two endo-crowns, that is, monobloc and endo-crown with primary abutment.

**Figure 2.**
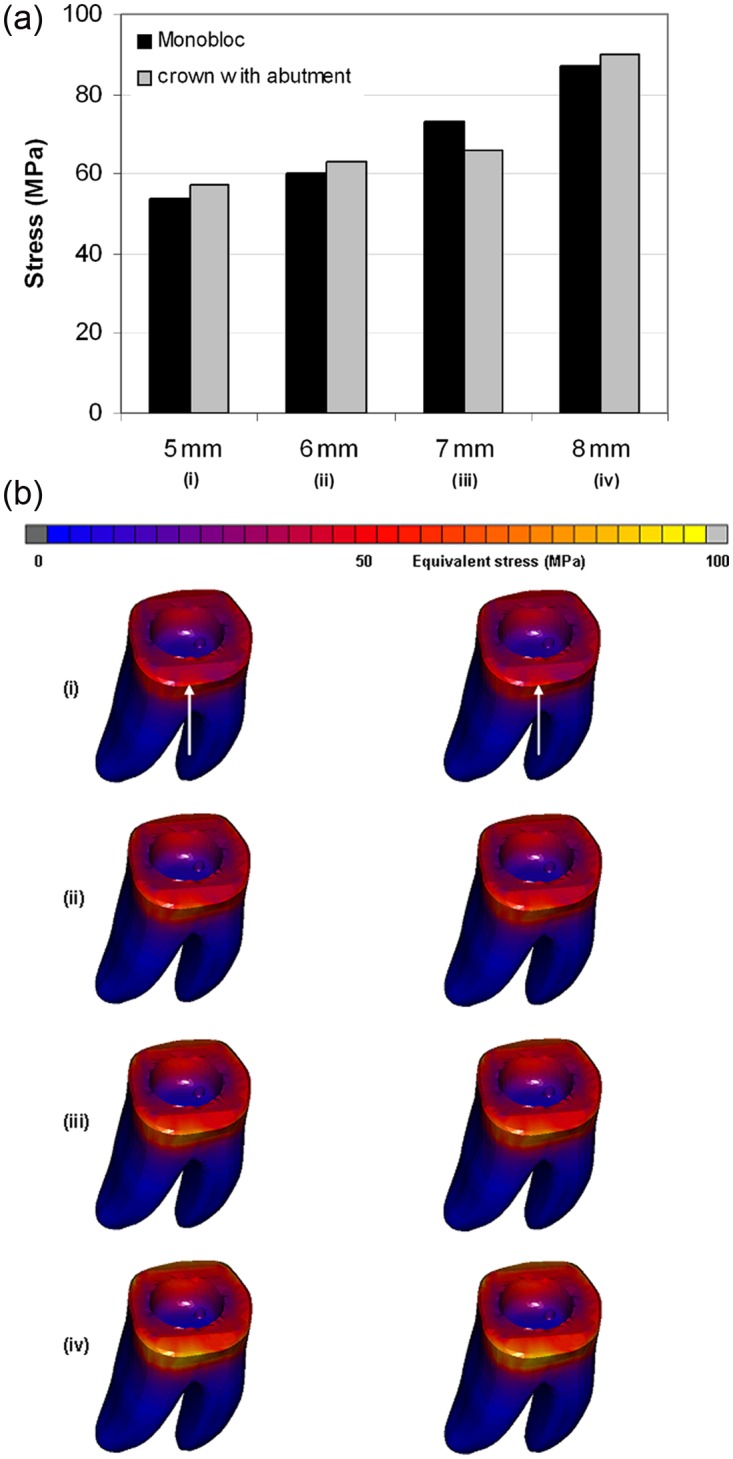
Total stress obtained after applying a static load of 1400 N on the endo-crowns at 5, 6, 7 and 8 mm distance from the finishing line: (a) the maximum obtained equivalent von Mises stresses and (b) equivalent von Mises stress distribution after applying the load at (i) 5, (ii) 6, (iii) 7 and (iv) 8 mm of the two endo-crown models. Monobloc (on the left) and endo-crown with primary abutment (on the right). The arrow indicates loading direction.

**Figure 3.**
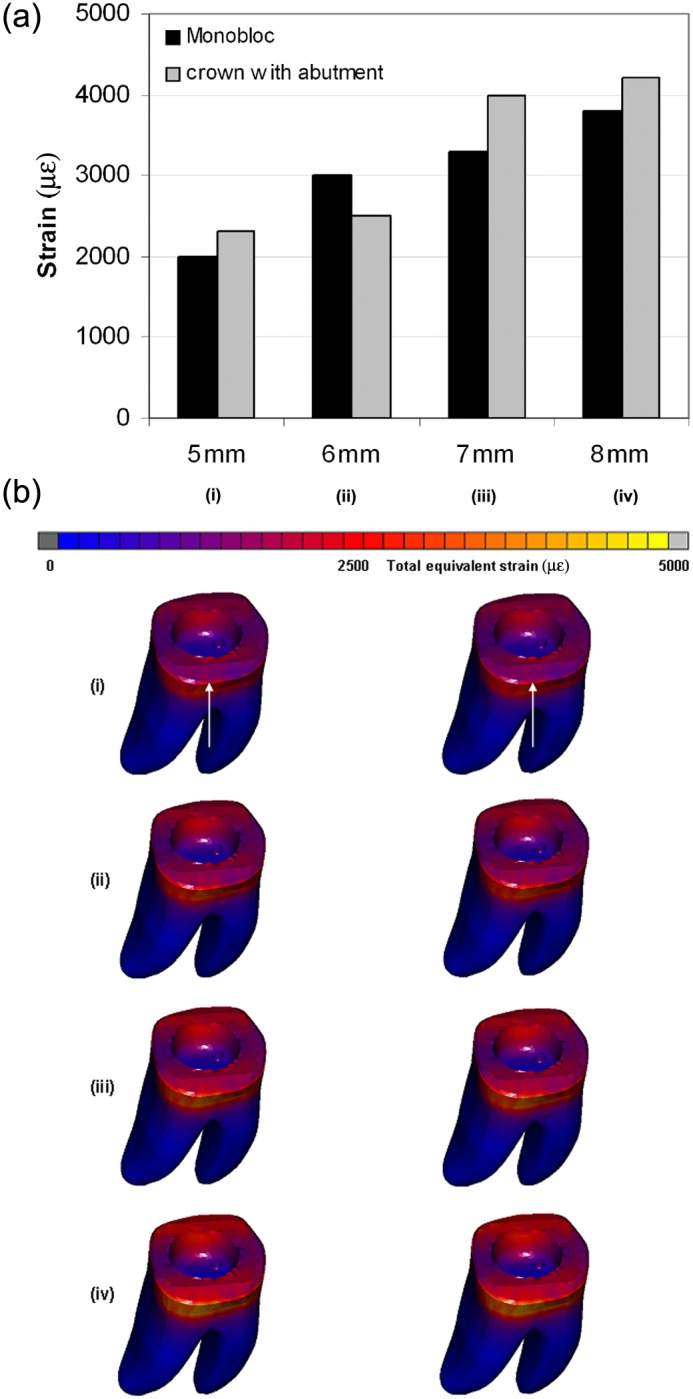
Total equivalent strain obtained after applying a static load of 1400 N on the endo-crowns at 5, 6, 7 and 8 mm distance from the finishing line: (a) obtained maximum total equivalent strains and (b) distribution of total equivalent strain after applying the load at (i) 5, (ii) 6, (iii) 7 and (iv) 8 mm of the two endo-crown models. Monobloc (on the left) and endo-crown with primary abutment (on the right). The arrow indicates loading direction

The stresses and strains were observed to be distributed over a larger area by applying the load on a higher point from restoration–tooth junction (Figures 2 and 3). The highest equivalent stresses and strains were concentrated at the restoration–tooth junction on the side of load application (Figures 2(b) and 3(b)).

### Endo-crowns

For both endo-crowns, the distribution of equivalent von Mises stress and total equivalent strain was similar when the force was applied at the same position. The magnitude of the endo-crown displacement was comparable for both endo-crowns too. Nevertheless, changing the height of the applied load showed different results.

**Figure 4.**
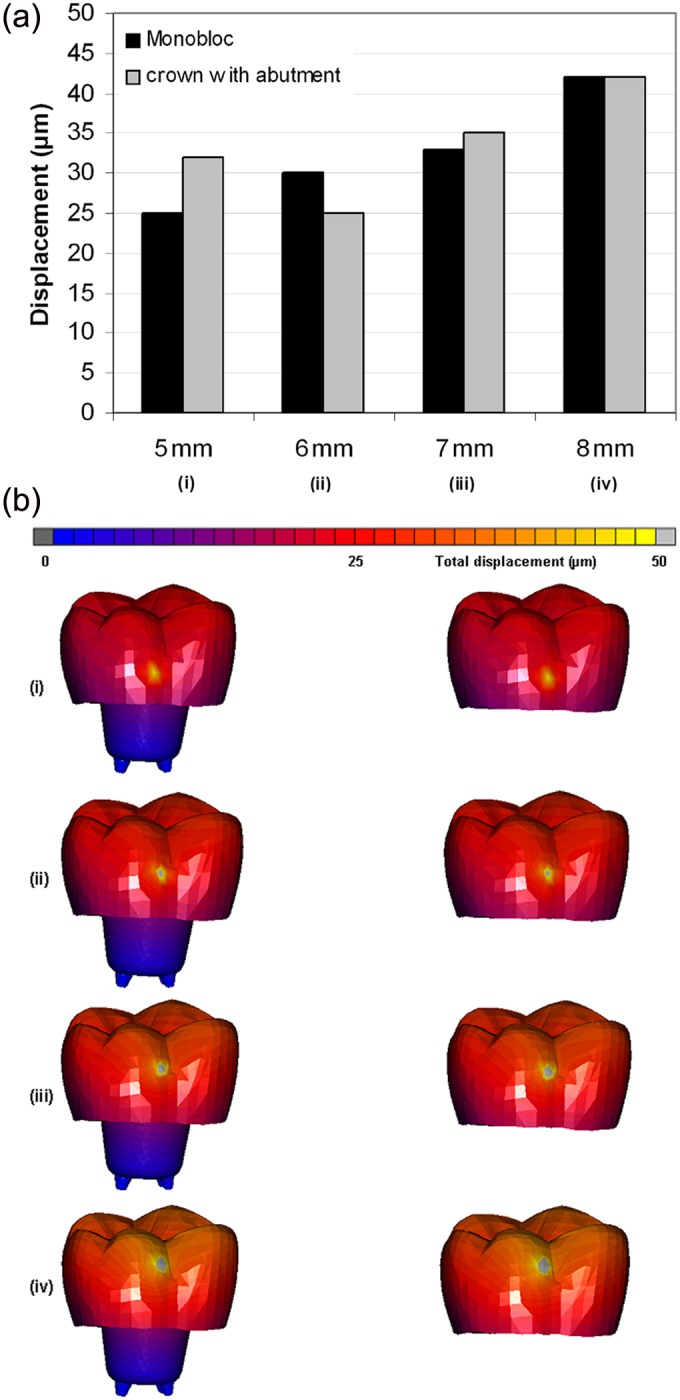
Obtained total displacement of the endo-crowns after applying a static load of 1400 N at 5, 6, 7 and 8 mm distance from the finishing line: (a) obtained maximum displacements and (b) displacement after applying the load at (i) 5, (ii) 6, (iii) 7 and (iv) 8 mm of the two endo-crown models. Monobloc (on the left) and endo-crown with primary abutment (on the right).

**Figure 5.**
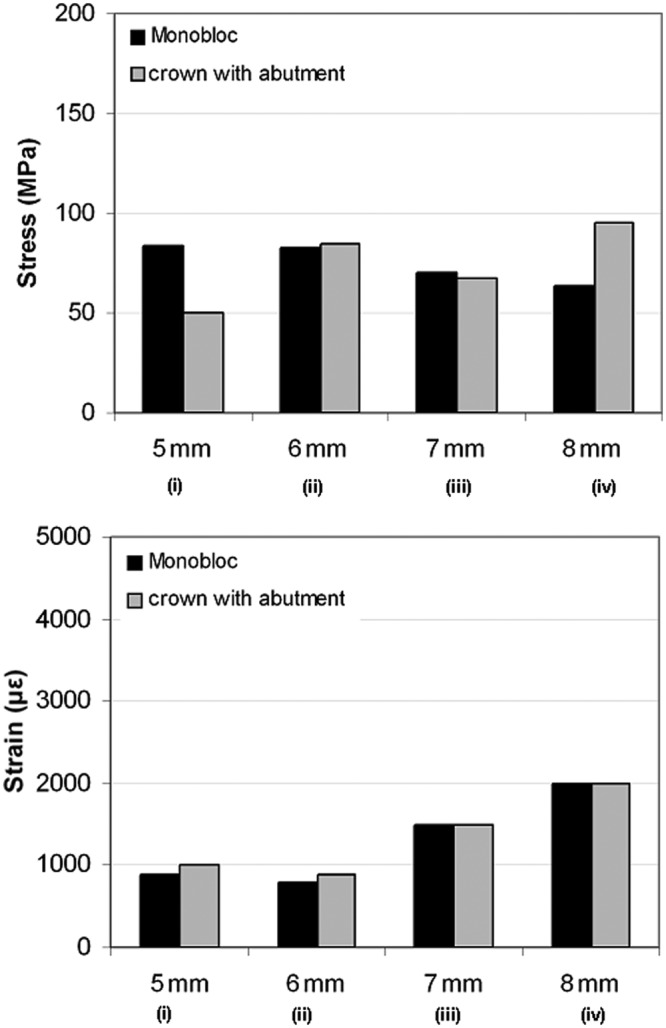
Maximum equivalent von Mises stress and total equivalent strain calculated for the endo-crowns. Equivalent von Mises stress and strain were considered in the presentation of the results.

**Figure 6.**
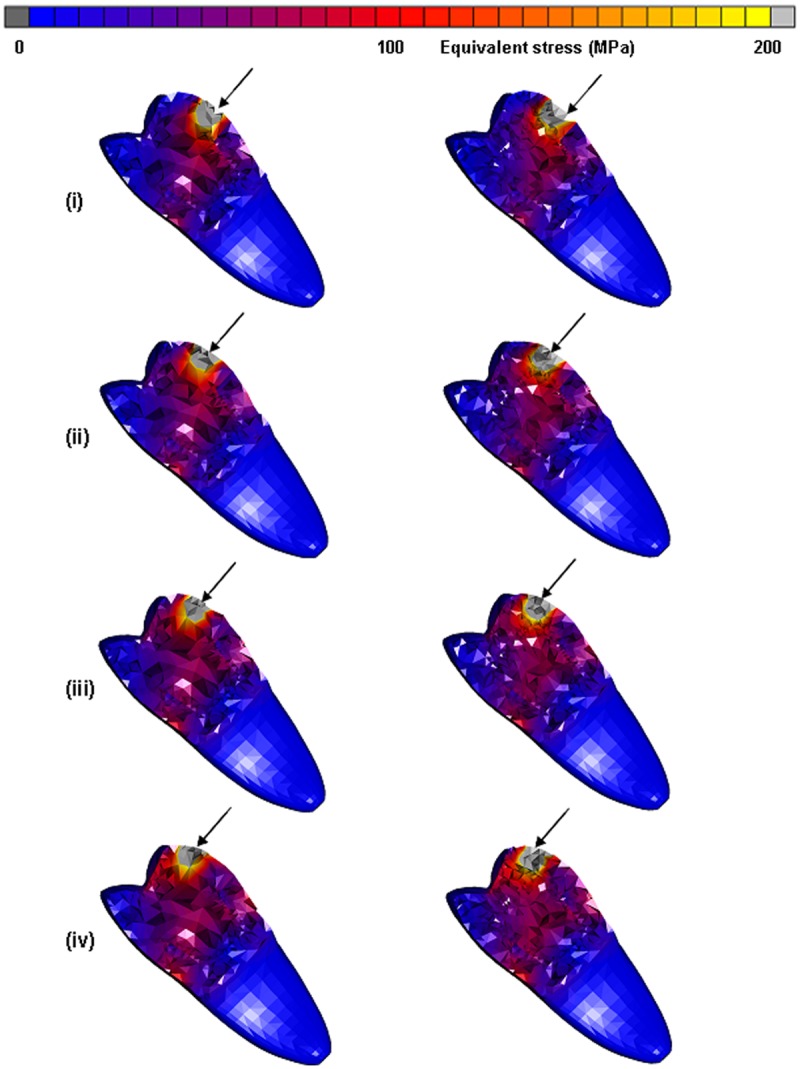
Obtained distribution of equivalent von Mises stress of the endo-crowns after applying a static load of 1400 N at 5, 6, 7 and 8 mm distance from the finishing line. Equivalent von Mises stress after applying the load at (i) 5, (ii) 6, (iii) 7 and (iv) 8 mm of the two endo-crown models. Monobloc (on the left) and endo-crown with primary abutment (on the right).

Endo-crowns had a minimal displacement (25–32 µm) when the force was applied at a height of 5 mm from the restoration–tooth junction, while the crown displacement increased to 25–30, 33–35 and 40–42 µm by moving the loading point to 6, 7 and 8 mm, respectively (Figure 4). The highest displacement was observed at the occlusal third of the endo-crowns (Figure 4(b)). Moreover, applying the loading at 5 mm height resulted in equivalent von Mises stress of 50–84 MPa and total equivalent strain of 900–1000 µϵ, while at 6, 7 and 8 mm, the equivalent von Mises stresses were 83–85, 67–70 and 63–95 MPa and the total equivalent strains were 800–900, 1300–1500 and 2000–2100 µϵ, respectively (Figure 5). However, the distribution of equivalent von Mises stress and total equivalent strain was wider with increasing the loading point to 8 mm. Figure 6 illustrates the distribution of the equivalent von Mises stress for the two endo-crowns at the four different loading points.

## Discussion

In this study, the mechanical behaviour of endo-crowns was investigated numerically by applying a load at four different heights from the restoration–tooth junction. Moreover, the difference between two construction concepts of endo-crowns was mechanically investigated from an engineering point of view. First, the 3D FE model of a lower first molar was developed that received an endo-crown as a monobloc. Second, another identical model was developed in which the endo-crown consisted of a primary abutment and a full crown. The targets of this study were based on a previous in vitro study that had investigated the above-mentioned two endo-crowns at two different heights of the load application (5 and 8 mm).

Tooth fractures may occur due to a single dynamic load or as a result of stresses that exceed the strength of the hard tissues or restorative replacement. Although possible, these forms of fracture are rather uncommon. Mechanical failures of a restored tooth are much more likely to result from fatigue, which is a cumulative process consisted of damage initiation and/or propagation. In the restored teeth, fatigue and fatigue failures are expected to result from cyclic loads that are associated with typical oral activities. In fact, fatigue crack growth within dentine has been cited as a major contributor to the failure of teeth with restorations. The fatigue strength for human dentine was reported to be between 25 and 45 MPa.^34,35^ The previous in vitro studies^29,30^ recommended to preserve as much as possible of the height of the prepared crown and not extend the preparation to the cementoenamel junction to avoid the overloading of the endo-crowns. In this study, the resulting equivalent von Mises stress within the remaining tooth structure was 54–57 MPa when the load was applied at the height of 5 mm and increased up to 87–90 MPa when the load was applied at the height of 8 mm from the tooth–restoration junction. The obtained equivalent von Mises stresses from the first loading condition (5 mm) was above the suggested strength of human dentine. These results are in agreement with the in vitro ones. The equivalent von Mises stress was more concentrated at the cervical remaining portion of the natural crown when the load was applied on the highest point (8 mm) than on the lowest point (5 mm, Figure 6). The same behaviour was observed for the total equivalent strain; 2000–2300 µϵ was obtained when the load was applied at height of 5 mm and 3800–4200 µϵ for the loading height of 8 mm. Such results could be expected as the loading point for the endo-crown was close to the junction to the tooth where the sudden jump in the mechanical properties of the restoration and dentine exists.

Zarone et al.^36^ reported that equivalent von Mises stress concentration in maxillary central incisors restored with an endo-crown is at the interface according to a 3D FE analysis. The interfaces of materials with different elastic moduli result in a weak point of a restorative system, because the stiffness mismatch of different materials influences the distribution of equivalent von Mises stress. Differences in the elastic moduli among ceramic, adhesive cement and the dentine might cause a risk of root fracture. Newly developed materials with mechanical properties as similar as possible to those of natural tooth hard tissues may decrease the frequency of unfavourable root fractures.^37,38^ Using an endo-crown restoration presents an advantage of reducing the effect of multiple interfaces in the restorative system and thereby makes the restored tooth more similar to a monobloc.

A point of concern is the influence of adhesion at the interface between different materials on the distribution of equivalent von Mises stress. The weak bond strength between ceramic and resin cements could lead to non-homogenous distribution of equivalent von Mises stress especially at the crown margins that could eventually lead to cohesive fracture of the resin cement leading to microleakage and its undesirable consequences.^39^

The total displacement of the endo-crowns was higher for the endo-crowns with primary abutment (32 µm) than monoblocs (25 µm) when the load was applied at 5 mm height. However, it was quite interesting that neither the differences of the magnitude nor the distribution of equivalent von Mises stresses and total equivalent strains was seen for the two endo-crowns, that is, monobloc and endo-crown with primary abutment. These observations restrict the possible reasons of the high failure rate of the endo-crowns with primary abutment in comparison to the monoblocs in the previous in vitro study to the different level of the applied load that had been used (8 mm for endo-crowns with primary abutment and 5 mm for monoblocs). The reason behind selecting these two different loading points in the in vitro study was to achieve an optimal position of the embedded teeth onto the table of the loading frame.

It is noteworthy to mention that even though laboratory fracture strength tests do not reproduce intra-oral loading conditions, they offer a controlled environment for preparing and testing the specimens thus allowing for comparable evaluation of the variables under investigation.^40^ Several studies had evaluated the fracture strength of endodontically treated teeth, and direct comparison between these studies is very difficult due to the interaction of many variables such as the tooth shape, tooth age, tooth structure, storage conditions, preparation and restoration methods, material differences, loading technique and most importantly classification of failure localisation.^41–45^

This numerical investigation indicates that a possible failure of the endo-crowns is associated with the height level of the applied force on the crown rather than the construction concept of the endo-crown itself. Moreover, paying attention to the total height of the endo-crown (position of the finishing line) and the location of the occlusal contact with the apposing teeth is clinically essential.

One of the limitations of this study was the use of an idealised geometry of the molars, the lack of considering the individuality of the tooth and cavity preparation. Studying the relation of different tooth geometries and preparation accuracy to the success of the two different endo-crowns is important to be investigated as a next step.

## Conclusions

Load distribution in the remaining tooth is similar with a monobloc and an endo-crown with primary abutment. For both endo-crowns, the region of load application plays a major role for their survival. The closer the region of the applied load to the restoration–tooth junction, the more desirable is the distribution of the load in the rest of the restored tooth.
